# Optimal Planning and Management of Land Use in River Source Region: A Case Study of Songhua River Basin, China

**DOI:** 10.3390/ijerph19116610

**Published:** 2022-05-28

**Authors:** Yucong Duan, Jie Tang, Zhaoyang Li, Yao Yang, Ce Dai, Yunke Qu, Hang Lv

**Affiliations:** 1 Key Laboratory of Groundwater Resources and Environment, Ministry of Education, Jilin University, Changchun 130012, China; duanyc19@mails.jlu.edu.cn (Y.D.); tangjie@jlu.edu.cn (J.T.); yangyao18@mails.jlu.edu.cn (Y.Y.); daice19@mails.jlu.edu.cn (C.D.); quyunke1024@163.com (Y.Q.); lvhang19@mails.jlu.edu.cn (H.L.); 2 Key Laboratory of Water Resources and Water Environment, Jilin University, Changchun 130012, China; 3 College of New Energy and Environment, Jilin University, Changchun 130012, China

**Keywords:** land use optimization, water retention, land planning framework, watershed management, China

## Abstract

Adjusting land use is a practical way to protect the ecosystem, but protecting water resources by optimizing land use is indirect and complex. The vegetation, soil, and rock affected by land use are important components of forming the water cycle and obtaining clean water sources. The focus of this study is to discuss how to optimize the demands and spatial patterns of different land use types to strengthen ecological and water resources protection more effectively. This study can also provide feasible watershed planning and policy suggestions for managers, which is conducive to the integrity of the river ecosystem and the sustainability of water resources. A watershed-scale land use planning framework integrating a hydrological model and a land use model is established. After quantifying the water retention value of land use types through a hydrological model, a multi-objective land use demands optimization model under various development scenarios is constructed. Moreover, a regional study was completed in the source area of the Songhua River in Northeast China to verify the feasibility of the framework. The results show that the method can be used to optimize land use requirements and obtain future land use maps. The water retention capacity of forestland is strong, about 2500–3000 m^3^/ha, and there are differences among different forest types. Planning with a single objective of economic development will expand the area of cities and cultivated land, and occupy forests, while multi-objective planning considering ecological and water source protection tends to occupy cultivated land. In the management of river headwaters, it is necessary to establish important forest reserves and strengthen the maintenance of restoration forests. Blindly expanding forest area is not an effective way to protect river headwaters. In conclusion, multi-objective land use planning can effectively balance economic development and water resources protection, and find the limits of urban expansion and key areas of ecological barriers.

## 1. Introduction

Human needs for habitat and natural resources gradually reshaped the earth’s surface. The interactions between land use change and climate, hydrological systems and biodiversity are having long-term impacts on global food supply, fresh water, forests, regional climate and atmospheric environmental quality [1,2,3]. These changes may be permanent or unstable, or may be short-term or long-term. They start from multiple locations on a small scale to large-scale regions, and eventually have a positive or negative impact on the global environment [4]. Different surface reflectance and warming characteristics of land surfaces such as forests and cities lead to different substance circulation of land surface–water and land surface–atmosphere, which affects the regional water vapor cycle, carbon cycle and vegetation growth [5,6,7]. Numerous studies have shown that human activities such as deforestation, farming, urban sprawl and water conservancy can change regional climate and precipitation distribution, and reduce the value and sustainability of ecosystem services [8,9,10,11]. In recent years, the macro-control of land has attracted the attention of local managers. Rational use of land resources limits the speed of urban expansion and ensures enough ecological functional areas are very effective methods.

It is well known that there is a strong interaction between watershed land and rivers. Land use and cover are the key factors affecting the river ecosystem, water quantity, water quality and aquatic biodiversity [12,13,14]. Land use change in the basin will lead to changes in surface roughness, plant transpiration and soil characteristics, and finally transform the existing water cycle balance into a new balance [2,4,15,16]. It is found that cultivated land expansion and deforestation will significantly affect the watershed water cycle by changing regional evapotranspiration [17]. In addition, deforestation reduces surface roughness, resulting in increased surface runoff [18,19]. For groundwater, the increase in impervious surfaces associated with urban areas lowers the water table [20,21,22]. Groundwater over extraction leads to the disappearance of springs worldwide, which affects the interaction between surface water and groundwater [23,24].

Numerous studies have proved that forest ecosystem has a strong ability to conserve water and ensure water quality, which shows important value in regulating climate, reducing flood peaks and alleviating soil erosion in most areas [19,25,26]. The upstream water source area of the river plays an irreplaceable role in ensuring the downstream water volume, so the water source area is an important ecological function area for water resources protection. Therefore, quantifying the ecological value and water retention value of different land uses, such as forests, farmland and wetlands, has become a topic in recent years. [27,28,29,30]. Water retention value estimation methods include small-scale field experiments [31,32,33] and large-scale evaluation and prediction, which mainly rely on remote sensing technology and hydrological models. In general, the research direction is gradually changing from discussing the internal mechanism of soil water retention to quantifying regional soil and water retention capacity [34,35]. Furthermore, based on quantitative ecological and water resources development goals, regional demand and spatial distribution of land use can be gradually optimized. However, pressure on ecological retention also limited economic development of the cities in the water source region, particularly in the mountains. Although some regions have introduced watershed management policies and provided ecological compensation funds for the water source area, the supports were still limited. In sum, the watershed planning should focus on quantifying the value of land use to water security, designing protection area accurately so that the land with high ecological value will not be destroyed.

Land use demands are often restricted by local economic development, population migration and policy orientation, while the spatial optimization of each land use type is affected by historical evolution, elevation, slope, climate, industrial layout, etc. [36]. The allocation of land demand is a plan to improve the efficiency of land resource use in the whole region by optimizing the allocation of different land use types based on multiple development objectives under the restriction of regional land use schemes [37]. Commonly implemented land use demand optimization methods include the time series analysis method, the Markov chain, the simulated annealing method, the genetic algorithm, etc. [38]. Combined with the spatial information management function of GIS, the optimization of the spatial pattern of land use can be realized, and the integration of multiple algorithms is the research direction [39]. The PLUS (patch-generating land use simulation) model integrates the land expansion analysis strategy and CA model based on multi-type random patch seeds (CARS), and completes practical research on landscape dynamic change in multiple study areas [40,41,42,43]. Therefore, this model has a great advantage in simulating landscape patch changes of natural land types concerned with landscape pattern changes.

This study attempts to build a land use optimization framework on the watershed scale to form a feasible future land use spatial pattern that can ensure the optimal economy, ecology and water sustainability of river source regions. In order to achieve this goal, Songhua River, the most important river in Northeast China, which ensures the water security of the two downstream provinces, is selected as the research area. The region has high forest coverage and abundant water resources, but its economy is underdeveloped, namely, it is often difficult to balance urban expansion and economic development. Therefore, this work aims to solve the following issues: (1) quantifying the amount of water retention through the SWAT (Soil and Water Assessment Tool, https://swat.tamu.edu/, accessed on 20 January 2022) hydrological model; (2) forming a multi-objective optimal allocation model of land use demand to promote economic development and protect ecological value and water conservation; (3) using the land use change simulation model to form the spatial planning of future land use development. Prospectively, it can provide reference for quantifying the amount of water retention and putting forward a multi-model coupled land use planning framework, and provide the government with reasonable planning for watershed management, economic taxation and future development.

## 2. Materials and Methods

### 2.1. Methods

**Figure 1:**
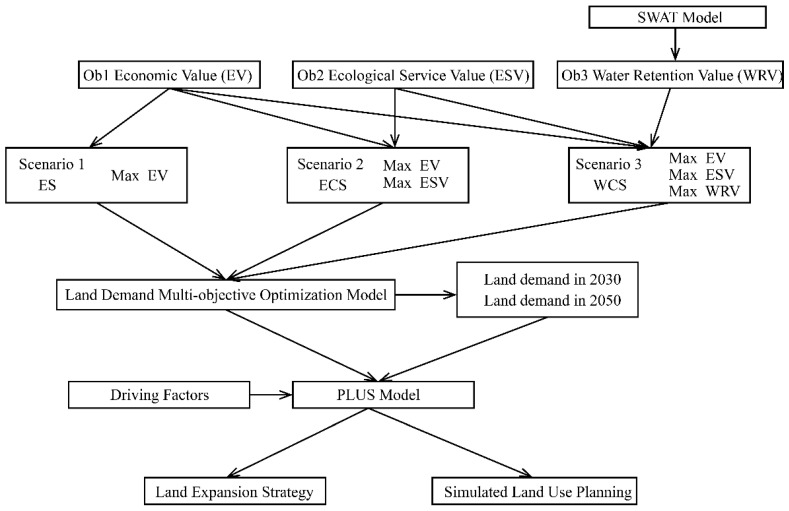
Land use planning framework and flow chart.

This study constructs a framework suitable for regional land use planning with high demand for water conservation. Figure 1 shows the flow chart of the steps followed in this study. We aim to provide managers with a way to develop the regional economy and ensure water security by forming an optimized land use plan for the headwaters of the river.

#### 2.1.1. Water Retention Value (wrv) Calculation by SWAT

In this study, the water retention amount can be obtained more accurately by constructing a watershed hydrological model. SWAT has been applied worldwide to analyze the temporal and spatial variation characteristics of hydrological processes such as surface runoff, evapotranspiration, soil water, and groundwater. The spatial database and attribute database of the SWAT model mainly include land use data, DEM, soil attribute data, meteorological data, and hydrological data. The meteorological and hydrological data are organized into the standard format required by SWAT and then input into the model. The observed runoff of the hydrological station is input into the calibration model SWAT-CUP software (USA, https://swat.tamu.edu/software/swat-cup/, accessed on 10 March 2022) dedicated to the SWAT model for parameter calibration and error analysis. The specific model construction and calibration process refer to the official description and related papers [44,45]. The accuracy of the hydrological model is usually measured by NS (Nash–Sutcliffe efficiency coefficient) and R^2^ (correlation coefficient), and the closer to 1, the better the simulation result. After constructing the SWAT, the water retention amount was calculated based on the water balance equation of the model, which served as the basis for assessing the water retention value of different underlying surfaces in the framework. The equation is as follows:

(1) Δ S=PREC−SURQ−ET−LATQ−GWQ

where ΔS is the change of soil water content in the time step; PREC is the precipitation; SURQ is the surface runoff; ET is the evapotranspiration; LATQ is the water quantity entering the aeration zone through the soil profile; GWQ is the underground water quantity. Next, introduce the water yield variable,

(2) WYLD=SURQ+LATQ+GWQ

where WYLD is water yield, i.e., the total amount of the water entering river runoff. The SWAT model divides the watershed into hydrological response units (HRU), and then the formula is converted into:

(3) $$W_{i}=\sum_{j=1}^{n}\left({\mathrm{PREC}}_{\mathrm{ij}}{-\mathrm{ET}}_{\mathrm{ij}}{-\mathrm{WYLD}}_{\mathrm{ij}}\right){\times A}_{\mathrm{ij}}\times 1000$$

$W_{i}$ is the water retention amount of land use i, m^3^; ${\mathrm{PREC}}_{\mathrm{ij}}$, ${\mathrm{ET}}_{\mathrm{ij}}$, ${\mathrm{WYLD}}_{\mathrm{ij}}$ are the PREC, ET and WYLD of the j HRU (hydrological response unit) of land use i, mm; $A_{\mathrm{ij}}$ is the area of the j HRU of land use i, km^2^; n is the number of HRU of land use i.

(4) $${\mathrm{WL}}_{i}=\frac{W_{i}}{\sum_{j=1}^{n}A_{\mathrm{ij}}}\;\times \;100$$

${\mathrm{WL}}_{i}$ is water retention amount per unit area of land use i, m^3^/ha. The water retention values of each land use type is calculated by the following formula:

(5) $${\mathrm{wcv}}_{i}{=\mathrm{WL}}_{i}{\;\times \;p}_{w}$$

where ${\mathrm{wcv}}_{i}$ is the water retention value of land use i, 10^4^ CNY (China Yuan)/ha; $p_{w}$ is the average water price for all administrative units in the catchment, CNY/m^3^, in this case the value is 3 CNY/m^3^.

#### 2.1.2. Multi-Objective Land Use Demand Optimization

First, we establish an optimal allocation model for land resource demand, including variables, objective functions, constraints and required parameters, with 2030 and 2050 as the target years. Land use according to the research goals, three land use planning objectives of economic benefit (EV), ecological service value (ESV) and water retention value (WRV) are set, and scenarios are proposed according to the different combination of these three objectives:

(1) Economic development scenario (ES), which only focuses on a single economic development goal, of course, within a certain development range, to maximize the EV objective function;
(2) Ecological protection development scenario (ECS), which maximizes two objectives of EV and ESV and attaches equal importance to sustainable economic development and ecological protection;
(3) Water resource conservation scenario (WCS), which simultaneously maximizes the three objectives of EV, ESV and WRV in order to strengthen the water retention capacity.

Second, the land use types are divided into ten categories, each represented by a code to simplify the following presentation. The area of each land use type is a variable in the optimization model. The details and the initial value of the area of each land use type are shown in Table 1. Third, for the parameters in objective functions, the economic benefits (ev), ecological service values (esv) and water retention values (wrv) of different land use types per unit area were required. Among them, the ev parameter is obtained by dividing the output value of forestry, agriculture, animal husbandry, fishery, secondary and tertiary industries from the local statistical yearbook by the area of the land use type corresponding to this function. The esv parameter is the value of ecological services provided by different land use types per unit area, which is determined by the value of China’s ecosystem services [46]. The wrv parameter is obtained in Section 2.1.1.

The formulas are shown in Table 2. Fourth, the constraints are based on multiple factors, such as current and future policy direction and long-term population change, which mainly include total area constraint, forest cover constraint and the upper and lower limits constraint of each land use type. Determining the upper and lower limits of each land use area in 2030 and 2050 was mainly based on the predicted value of the Markov chain, and also referred to the limit of the minimum agriculture land area, urbanization rate, and per capita living land area in the study area. The constraint range in 2050 is larger than the constraint in 2030 to ensure a relatively better objective function value in a larger optimization space. The constraint conditions of the optimization model are described in detail in Table 3. Variables, objective functions, and constraints constitute the land use demand optimization model, and the next step is to solve the model. Among the three scenarios, the ES scenario is a single objective problem, which is solved by linear programming; the others are multi-objective problems solved by NSGA-II [47]. The procedure code is written in Python language (https://www.python.org/, accessed on 2 July 2021). The main genetic algorithm process relies on Geatpy2.6.0 (http://www.geatpy.com/, accessed on 2 July 2021), a high-performance and practical evolutionary algorithm toolkit in Python. It provides a convenient, modular, problem-solving-oriented evolutionary algorithm framework. Since the initial population is randomly generated in the algorithm, the Pareto optimal population generated after each iteration is reached is different. In order to determine the appropriate number of iterations and avoid falling into local optimization, seven groups of tests are set, the number of iterations is at least 1000 times, each group is increased by 1000 times, repeated three times, and the top 200 in the ranking are output. If the value of the objective function changes significantly, the number of iterations may be insufficient, there may be a possibility of falling into local optimization, and the number of iterations needs to be increased. The top five ranked are selected to form the optimal group, in which the individual closest to the current situation is selected as the land demand value of the scenario. Through the above steps, the area demand of various land use types in future years under different scenarios can be obtained, which is also the input data for PLUS to obtain future land use maps.

#### 2.1.3. Land Use Change Simulation and Prediction Land Use

The PLUS model is available to simulate the change of land use patches and to analyze the drivers of land use change. The PLUS V1.2 5 used in this research is provided on github (https://github.com/HPSCIL/Patch-generating_Land_Use_Simulation_Model, accessed on 10 July 2021). The input data for PLUS are land use maps and maps of potential development factors for the initial year and the simulation year. The error between the simulated land use map and the real map can be reduced by adjusting parameters of the model, and the error is measured by Kappa coefficient and figure of merit (FOM) value. Simultaneously, contributions of all the driving factors for the expansion of each land use type can be obtained. In this case, we take 2010 as the initial year and 2020 as the simulation year. A total of 15 potential development factors are selected to simulate the development probability of different land types, including natural condition factors such as temperature, precipitation, terrain, slope and soil type, and urban development factors such as population, GDP, distance from different grades of highways and government. According to the characteristics of the mineral water and tourism industry in the study area, the spring with a flow of more than 1000 m^3^/d and tourist attractions are also considered as driving factors. In addition, the evolution of landscape pattern will affect the ecological service value. Landscape metrics are not only the driving factors leading to the habitat heterogeneity in the residual forest debris but also the index guiding the conversion of farmland to forest and ecological restoration. Therefore, this research selects 6 class-level metrics and 17 landscape-level metrics to explore the evolution of landscape patterns from 2010 to 2020, and detailed information of landscape metrics [48] is shown in Table A1. At the same time, it can also characterize and quantify the similarity of landscape characteristics between simulation results and observation results. If all the indicators to measure the simulation accuracy, including statistical indicators and landscape indicators are within the acceptable range, the predicted future land use map can be obtained by entering the demand for the land use type in the future years obtained in the previous step. The Moran’s I [49] of global autocorrelation and the LISA (local indications of spatial association) cluster map [50] are used to evaluate the spatial distribution pattern and aggregation of total ecosystem service value and water retention value in the past ten years and the planned future 2030 and 2050 at the patch level.

### 2.2. Study Area

**Figure 2:**
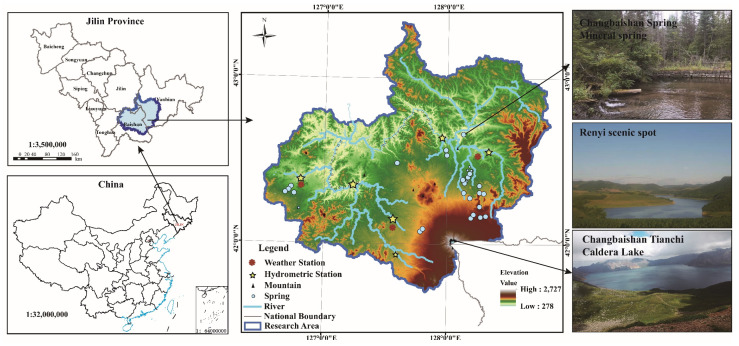
The location of the research area. DEM is downloaded from http://www.gscloud.cn/sources/accessdata/310?pid=302, accessed on 5 June 2021. The map of China was downloaded from http://bzdt.ch.mnr.gov.cn/, accessed on 5 June 2021, GS (2019)1676.

In order to explore the applicability of the planning model to the land use planning of important forest ecosystems and water retention and protection areas, the source area of Songhua River in the Changbai Mountain Area of China is selected as the study area. Figure 2 shows the location and terrain. Changbai Mountain is the highest mountain system on the eastern edge of Eurasia, with a complete forest ecosystem. It is not only a “hot spot” of global biodiversity but also the main water source of the Songhua River, an important river in Northeast China. The source area of the Songhua River supplies 39% of the water of the Songhua River, ensuring the water safety of tens of millions of people in the two downstream provinces, and has an extremely important ecological status and water retention value. The area is a typical volcanic landform, covered with lava flows (basalt and trachyte) [51], with an altitude of about 1000–1800 m. The climate conditions are temperate continental mountain climate affected by the monsoon. The average annual temperature is between −7 and 3 °C, and the annual precipitation is 700–1400 mm (http://data.cma.cn/). The precipitation from June to September accounts for 60–70% of the annual precipitation. Most of the groundwater is basalt hole fissure water, which is discharged in the form of evaporation and spring. The spring flow is voluminous, the largest can reach more than 30,000 m^3^/d, and it is rich in metasilicic acid, attracting some mineral water companies to invest and build factories here. Contrary to the contradiction that the economic development of other developed regions destroys the regional ecology, due to its unique ecological value and tourism resources, the economic development and the development of land resources in this region are seriously limited. It has always been a poor area with slow economic development and insufficient government tax revenue. Therefore, how to develop the region’s economy through planning for future land use needs and find out the best solution between protecting forest ecology and water sources is a difficult problem.

### 2.3. Data Source

The data required for this study are divided into three categories: spatial database and meteorological data necessary for the construction of SWAT hydrological model; land use status and future government planning data required for multi-objective land demand planning; and economic development data and land use grid data necessary for the construction of PLUS model, spatial natural conditions and the social factor driving factors. Table 4 shows the specific data sources and uses. Among them, the land use data are superimposed from the two data sources, GlobeLand30 V2020 and global CCI-LC land use maps, and further calibrated based on satellite remote sensing images. Due to the wide distribution of forests in the study area, the forest land is subdivided into deciduous broad-leaved forest, evergreen coniferous forests, needle-broad-leaved mixed forest, and open forest, which can discover the changes of different forest types.

## 3. Results

### 3.1. Accuracy of SWAT Model and Water Retention Value

**Figure 3:**
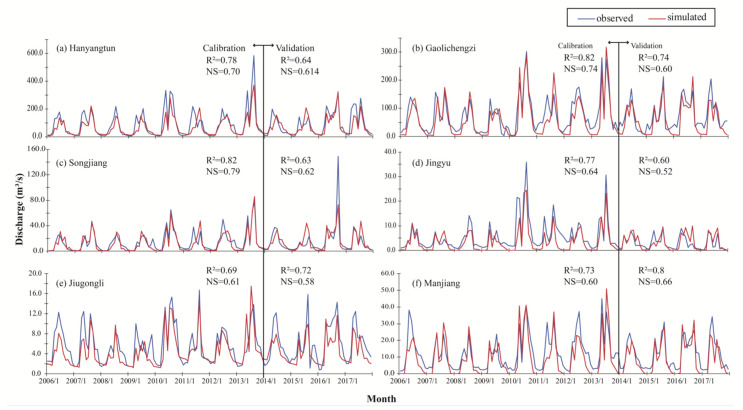
Observed and simulated discharge for six monitoring points and NS and R^2^ values in calibration and validation periods.

**Figure 4:**
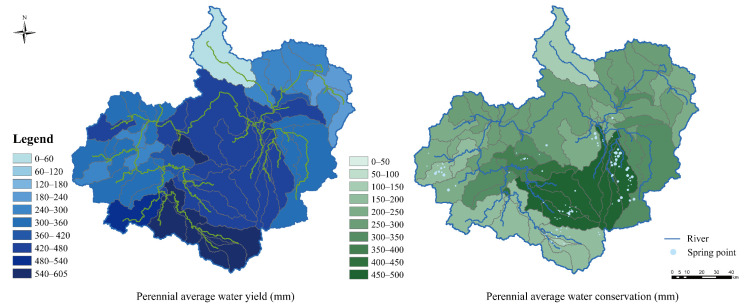
Spatial pattern of annual average water yield amount and water retention amount.

The study area is divided into 63 sub-watersheds. The parameters of the model were calibrated using the monthly average runoff of the six hydrological stations shown in Figure 1. The calibration period is 2006 to 2014; after that is the validation period. The simulation results and accuracy are shown in Figure 3. During the calibration period, the NS value of the runoff at all hydrological stations was greater than 0.6, and during the verification period, all except Jingyu and Jiugongli hydrological station were above 0.6, so the simulation results were acceptable [44]. Therefore, the water balance equation output from the SWAT model can be used to estimate the WL_i_, which is the water retention amount per unit area according to Equations (1)–(4). The results are shown in Table 5. The water retention capacity of forest land is the highest, and coniferous and broad-leaved mixed forest and deciduous broad-leaved forest are higher than coniferous forest, followed by wetland and shrub forest. The water retention capacity of sparse forest land, cultivated land and grassland is relatively low. The construction of urban hard pavement and drainage pipe network weakens the city’s ability to retain precipitation. From 2006 to 2017, the model results show that the average precipitation in the study area was 754 mm/a, of which the annual average water production and annual average water storage accounted for 51% and 38%, respectively. The results obtained are similar to those of previous research [52,53]. In general, the plant and soil conditions give the area a strong water retention capacity and sufficient groundwater recharge capacity. Figure 4 shows the average annual amount of water yield and water retention for 63 sub-basins. From the perspective of spatial distribution, the southern mountainous area of the study area has a large water yield and a high supply of surface water sources to river runoff. The central part of the study area has strong water retention capacity, with an average annual water retention capacity of 390–490 mm/a, which is very similar to the distribution of mineral springs in the study area. In total, 58% of the springs with flow rates greater than 5000 m^3^/a are concentrated in this part of the region. Therefore, this area is the area with the strongest water resources storage capacity and the most frequent recharge to surface runoff, underground runoff and spring water.

### 3.2. PLUS Model Simulation Accuracy and Results

#### 3.2.1. Model Accuracy and Landscape Comparison

A random sampling method is adopted, and the sampling rate is set to 5%. In order to prevent overfitting, 10 regression trees were developed. The value of the neighborhood effect is 3. The patch generation decay threshold and expansion coefficient are set to 0.9 and 0.1, respectively. During the calibration process, it is found that due to the slow economic development in the study area, the land use change in the past 10 years is small, and 80% of the land in the study area is forest land, which also has high requirements for the future forest coverage. In particular, overfitting should be avoided, and the number of regression trees should be appropriately reduced to reduce the expansion coefficient, to reduce the generation of new land use patches. The results show that the kappa coefficient and FOM values characterizing the simulation accuracy are 0.79 and 0.14, respectively, and the simulation results are acceptable [40,41].

In addition, we also used the selected landscape metrics to quantify the similarity between the simulation landscape and the observed landscape of 2020. The landscape pattern metrics of class-level and landscape-level are shown in Table A2 and Table A3. In the past ten years, the landscape pattern index of each land use type in the study area remained stable. From Table A2, it can be discovered that the forest patches changed little, but the aggregation index AI of the sparse woodland patches increases. The patch density (PD) of grassland landscape decreased. The landscape shape index (LSI) that indicates the patch shape increased significantly in urban patches, which is mainly affected by urban expansion. The LSI of wetland patches increased and the AI aggregation index decreased, which may be affected by precipitation and human activities. From Table A3, the landscape fragmentation index decreased slightly, and the diversity index SHDI and SHEI did not change significantly. The similarity of the landscape pattern indicators between the simulation results and the observation results is high, but the ratio of the indicators representing landscape fragmentation and shape to the observation results is relatively large, especially for forests. The reasons may be related to the large forest landscape dominance, the high degree of patch aggregation and the small land use change in the study area. It may be that the changes in forest patches during the formation of new patches are over-simulated.

#### 3.2.2. Analysis of Land Use Expansion Strategy

**Figure 5:**
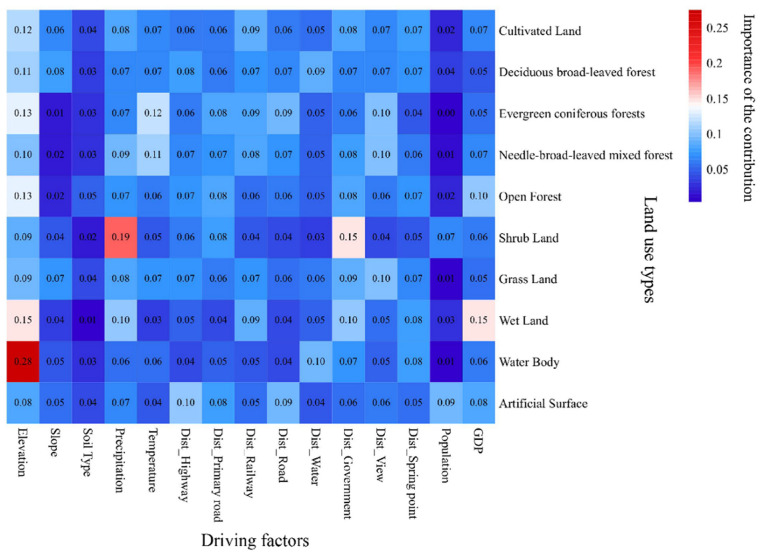
Heat map of the importance of the contribution of each driving factor to the growth of specified land use types.

The PLUS model can output the contribution of the set driving factors to the change of each land use type. Figure 5 shows the contributions of different driving factors in driving the other land uses to convert to a specified target land use type, and the numbers on the graph indicate the contribution value. The larger the value, the greater the contribution of this factor to the specific land use transfer direction compared with other factors. In order to measure the contribution level, we divide the contribution value into four levels, low contribution means that the contribution of the driving factor is very low, general contribution means that the contribution is not obvious, medium contribution means that there is a certain contribution, but it is not a dominant factor, and great contribution means that it has a strong influence. Table 6 is the classification standards and the number of contribution value at each level, $\bar{X}$ is the mean of all data and σ is the standard deviation. The contribution of most driving factors is between the general contribution and the medium contribution, and the number of high and low contributions is consistent. Among the 10 land use types, the expansion of 7 types is driven by elevation, with a large contribution, and the contribution to grassland, shrub, and construction land is medium, indicating that the terrain of mountainous areas has a significant impact on the initial form of regional land use, forest vegetation, water body, and wetland fluctuation. However, due to the slow speed of urban expansion in this area, the terrain has not seriously restricted urban expansion. The change path of evergreen coniferous forest and coniferous broad-leaved mixed forest is similar, and it is also affected by temperature and distance from scenic spots, which is related to the fact that the growth and distribution of coniferous forest are more vulnerable to climate change [54]. The scenic spots in the region are mainly mountain forest landscape, which is located in the zone with high altitude range 1000–1800 m and a large area of coniferous forest, resulting in an interactive relationship between the two. Both wetland and open forest land are driven by GDP. With economic development, the area of open forest land and wetland increases. The distance from the railway is the main driving factor for the expansion of building environment. Between 2010 and 2020, both GDP and road mileage increased by 20% in the study area. The development of towns and villages in mountainous areas depends very much on traffic conditions, and the railway is the basis for ensuring basic passenger transport, freight transport, and long-distance transportation. In particular, precipitation has a strong driving force on the change of shrub land, and the contribution of each driving force to grassland is similar.

### 3.3. Future Land Use Simulation

#### 3.3.1. Future Land Demand Simulation Based on the Multi-Objective Scenario

Table 7 shows the optimization results of multi-objective land use demand in 2030 and 2050 under the three scenarios, including the area of each land use type and the corresponding objective function value. The results show that under the single objective ES scenario, the optimal solution of linear programming with economic benefit maximization as the single objective is to maximize the area of AGRL and URBN with high economic benefit under the condition of ensuring the basic forest area. Under the other two scenarios, the cultivated land area decreases compared with 2020, which is 18 × 10^4^–19 × 10^4^ ha in 2030 and 14 × 10^4^–16 × 10^4^ ha in 2050. The urban area increases, which is 2.4 × 10^4^–2.7 × 10^4^ ha in 2030 and 2.8 × 10^4^–2.9 × 10^4^ ha in 2050. The urban expansion area of WCS is smaller than that of the ECS scenario. Compared with 2020, the total forest area remained stable, the forest type configuration changed, the open forest area decreased under ECS and WCS scenarios, and the WCS scenario decreased more, and the area of deciduous broad-leaved forest and coniferous broad-leaved mixed forest increased. The target value of water retention in the WCS scenarios is higher than that in other scenarios and is about one-fourth of the total ESV, which is consistent with the previous research. Overall, the three objective function values after the optimized configuration are better than 2020, which can be used as the data basis for spatial optimization. It also provides managers with alternative demand schemes for short-term and long-term planning, ensures water safety and mineral water supply, and forms a reasonable land use demand allocation for regional sustainable development.

#### 3.3.2. Future Land Spatial Pattern Simulation Based on PLUS

**Figure 6:**
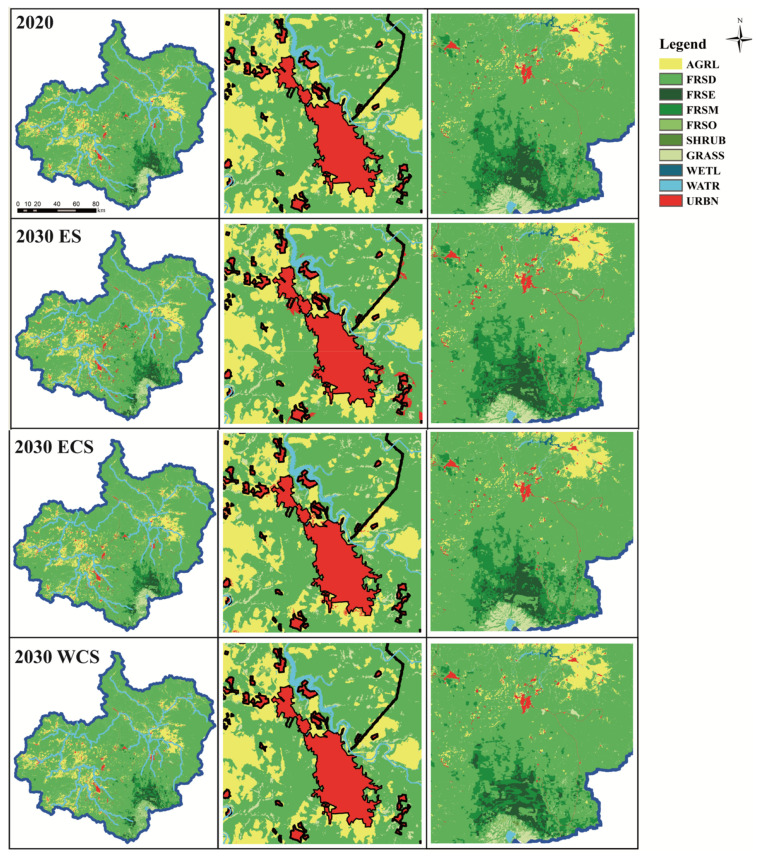
Land use planning maps of the three scenarios in 2030: the first column is land use map, the second column is the magnification of Fusong County, and the third column is the magnification of Changbai Mountain area. Each land use name code is the same as in Table 1.

**Figure 7:**
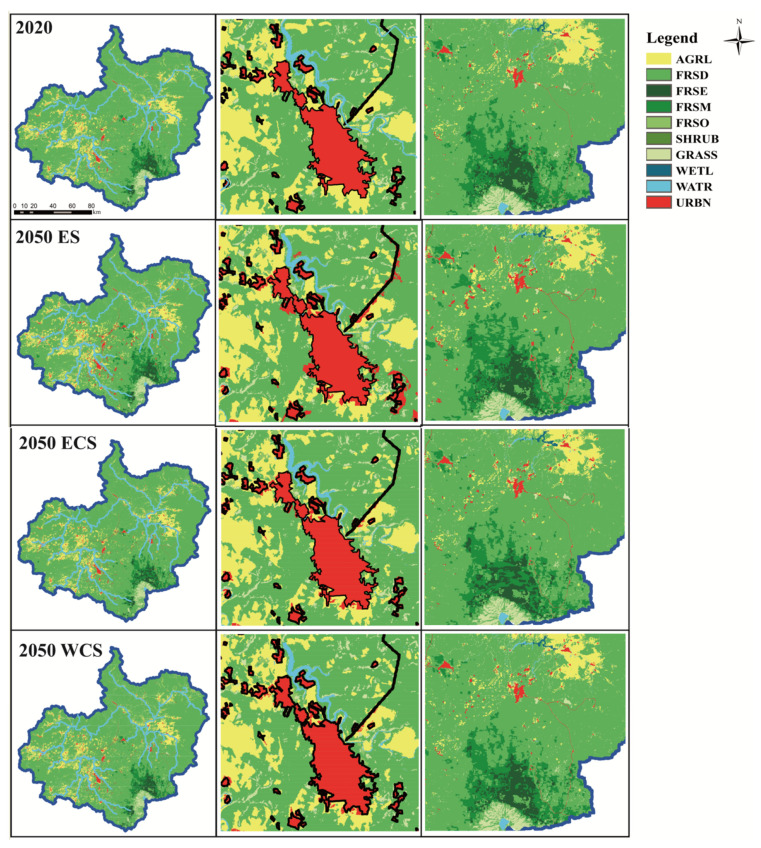
Land use planning maps of the three scenarios in 2050, the first column is land use map, the second column is the magnification of Fusong County, and the third column is the magnification of Changbai Mountain area. Each land use name code is the same as in Table 1.

The land use planning map in 2030 and 2050 is shown Figure 6 and Figure 7. On the whole, the changes of various land use types in 2050 are significantly more than that in 2030, and the land use conversion near the urban built-up area and around the Changbai Mountain Area in the southeast of the study area is more intense. The forest land has a spreading trend, and the area of high-altitude tundra grassland is reduced. In the ES scenario, dominated by economic value, the urban expansion is the most rapid. The cultivated land and deciduous broad-leaved forest around the town are transferred to the urban area, and the cultivated land is also expanding. In the ECS scenario, the land use conversion is small and the wetland water body increases. In addition, under the WCS scenario, the transformation among the four forest types is more obvious, and the open forest area is reduced, indicating that if water conservation is the policy orientation, more attention should be paid to the change and protection of forest types. Excessive pursuit of a large area of forest land is not a good management method, which may not only fail to achieve the desired result but also limit the development of the regional economy. It is very important to provide targeted protection for primeval forests and primary vegetation. Simultaneously, the selection of plant species and long-term maintenance of artificial forests should be strengthened. To summarize, in order to guarantee water resources through protecting the forest ecosystem, it is necessary to guarantee a certain forest coverage rate first, and then strengthen the optimization of forest vegetation quality, rather than simply expanding the area. Compared with the three scenarios, urban expansion in the ES scenario tends to occupy the surrounding deciduous forest land, while the other two scenarios are more inclined to occupy cultivated land.

**Figure 8:**
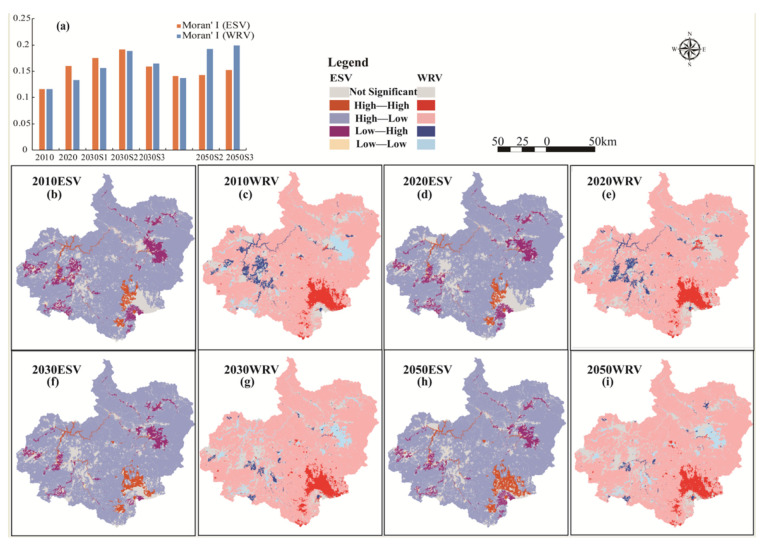
Spatial autocorrelation analysis of ESV and WRV from 2010 to 2050 of WRS scenario: (**a**) is the Moran’s I change diagram and (**b**,**d**,**f**,**h**) are the LISA maps of ESV; (**c**,**e**,**g**,**i**) are the LISA maps of WRV.

Figure 8 shows the spatial autocorrelation analysis map of ESV and WRV from 2010 to 2050. These two values show obvious clusters and differences in spatial distribution. Moran’s I is higher than 0.1, and the z value is much higher than 1.96, that is, the spatial distribution of total ecosystem service value and water retention value is a clustered pattern within a 95% confidence interval, with a strong positive correlation. In other words, patches with higher values tend to cluster together, which is more obvious in 2020 than in 2010, and this is consistent with the direction of the natural evolution of cities and ecosystems in the study area. Moran’s I under the three planning scenarios of 2030 and 2050 is significantly higher than that in 2010 and slightly higher than that in 2020. Compared with the three scenarios, the Moran’s I of the ES scenario is smaller. The LISA cluster map divides the region into four types, namely, HH and LL of a positive correlation type and LH and HL of a negative correlation type. Spatially, HH clusters of ESV and WRV are located in the primeval forest of Changbai Mountain in the southeast of the study area, LL clusters are located in the urban and farmland concentration areas in the East and West, and most areas are HL clusters. The area of LL clusters of WCS is larger than that of ESV. In terms of time, the area of LL cluster patches continued to increase from 2010 to 2050. Separately, the expansion of LL cluster patches of WRV is more significant than that of ESV. In summary, with social development, patches with low ecological value tend to aggregate and expand, and the change and development of land use may bring some ecological risks.

## 4. Discussion

For the accuracy of the model selected in this paper, the SWAT model has acceptable simulation accuracy for the hydrological cycle of this study and can be used as a reference to water resources management and future water resources prediction in mountainous areas, but there are also some deviations, especially in the snowmelt period and sub-basins with complex geological conditions where surface water and groundwater exchange are frequent. The coupled model of surface water and groundwater can be used to obtain more accurate water balance simulation results in the study area [55]. A large amount of spring water in this area is also a major factor affecting the simulation accuracy [51,56]. Especially in the dry season, some large springs contribute greatly to the runoff. Therefore, establishing a hydraulic connection between the dynamic flow of spring water and the river can also improve simulation accuracy. The PLUS model has high simulation accuracy for the landscape patches in the study area. It is necessary to deeply understand the basic characteristics of the study area, the history of land use transfer, and the policy guidance of managers so that models and parameters can be adjusted to suit different areas. Carefully select the driving factors and comprehensively consider the geographical conditions and industrial characteristics [57]. If the city grows fast or the land use policy changes suddenly, the simulation accuracy will be affected.

The land use planning framework guided by water resources protection constructed in this study can quantify the value of regional water retention, and the land demand and land use planning maps under different development scenarios can be obtained through the land use demand optimization model. On the whole, the results have credibility, and can effectively improve the objective function values of economy, ecology, and water retention through the optimization process. The obtained water conservation value is similar to the previous research results, which is reasonable [58,59,60]. However, due to the high restrictions on forest cover, slow urban development, and population loss, the optimization space is limited. Furthermore, many studies have also demonstrated that the increase in vegetation area may also reduce water supply and cause downstream water demand conflicts, especially in arid and ecologically fragile areas [61,62]. Therefore, scientific and reasonable land use development models should be constructed in different river basins. Expanding the forest area or using the same management model is likely to cause the ecological restoration results to be counterproductive. Furthermore, there is a strong water holding capacity in the middle of the study area, which is a lava flow unit with thick basaltic strata and formed joints and fissures. The surface is covered with a large area of coniferous and broad-leaved mixed forest. High forest vegetation coverage plays a key role in regional water resource retention and microclimate maintenance [63]. The soil is dark brown soil and argillaceous dark brown soil. These natural conditions lead to strong water retention capacity in the region, which can retain precipitation in soil and groundwater, maintain regional climate and water vapor cycle, and provide a continuous source of water for downstream surface runoff [64]. Therefore, in future planning, the area should be regarded as a key nature reserve [65], and the control of natural resources should be strengthened to avoid excessive urban expansion and the construction of high-polluting enterprises. At the same time, the quality and quantity of surface water and groundwater are regularly monitored, and restrictions are imposed on mineral water exploitation and water conservancy project construction.

## 5. Conclusions

This study forms a watershed-scale multi-objective land use planning framework oriented to ecological maintenance and water resources security. The framework uses the SWAT model and water balance to calculate the water retention of different land use types, and then builds a multi-objective land use demand optimization model aiming at maximizing economic value, ecosystem service value and water retention value. The PLUS (patch-generating land use simulation) model is applied to obtain the driving factors of changes in different land use types and simulate future land use maps under the economic development scenario (ES), the ecological protection scenario (ECS) and the water resource conservation scenario (WCS). The application results in the source area of the Songhua River indicated that the framework can obtain optimized land use planning. The results indicate that the forest land had the highest water retention capacity, and the mixed coniferous and broad-leaved forests are higher than the coniferous forests. The water retention value (WRV) is about one quarter of the total ecosystem service value (ESV). The PLUS model has high simulation accuracy and can be used to explore land use change strategies in the study area. Elevation has a significant effect on changes in forests, water bodies and wetlands, but does not currently limit urban sprawl. In addition, distance from railways is a major driver of construction land change. Urban expansion is obvious under the ES scenario, and the transition between different forest land types is the most prominent under the WCS scenario. In both the ECS and WCS scenarios, the total value of ESV and WRV in the basin in 2030 and 2050 is higher than in 2020. Spatially, patches with low ESV and WRV tend to cluster, which may cause ecological threats. In order to protect the forest ecosystem and the sustainable development of water resources, key protected areas should be designated and the forest type should be paid attention to.

## Figures and Tables

**Table 1:** Land use change of the research area from 2010 to 2020.

Land Use	Code	Variable Name	Percentage of Total Area (%)	
			2010	2020
Cultivated Land	AGRL	$x_{1}$	11.42	10.12
Deciduous Broad-leaved Forest	FRSD	$x_{2}$	78.13	79.01
Evergreen Coniferous Forests	FRSE	$x_{3}$	1.39	1.41
Needle-broad-leaved Mixed Forest	FRSM	$x_{4}$	3.07	3.04
Open Forest	FRSO	$x_{5}$	0.33	0.45
Shrub Land	SHRUB	$x_{6}$	0.02	0.02
Grass Land	GRASS	$x_{7}$	3.89	3.64
Wet Land	WETL	$x_{8}$	0.02	0.06
Water Body	WATR	$x_{9}$	0.99	1.00
Artificial Surface	URBN	$x_{10}$	0.75	1.27

**Table 2:** Objective function of land use demand optimization model. CNY: China Yuan.

Objective Function	Formula	Units
Economic value objective (EV)	$\mathrm{EV}=\overset{n}{\underset{i=1}{\sum }}{\mathrm{ec}}_{i}\;A_{i}$ $=1{.93x}_{1}+0{.03x}_{2}+0{.03x}_{3}+0{.03x}_{4}+0{.03x}_{5}+0{.03x}_{6}+1{.71x}_{7}+0{.59x}_{8}+0{.59x}_{9}+99{.57x}_{10}$	EV 10^4^ CNY ec_i_ 10^4^ CNY/ha A_i_ ha
Ecological service value objective (ESV)	$\mathrm{ESV}=\overset{n}{\underset{i=1}{\sum }}{\mathrm{esv}}_{i}\;A_{i}$ $=1{.17x}_{1}+4{.04x}_{2}+3{.09x}_{3}+4{.06x}_{4}+3{.37x}_{5}+2{.68x}_{6}+2{.12x}_{7}+11{.96x}_{8}+11{.96x}_{9}+1{.16x}_{10}$	ESV 10^4^ CNY esv_i_ 10^4^ CNY/ha A_i_ ha
Water retention value objective (WRV)	$\mathrm{WRV}=\overset{n}{\underset{i=1}{\sum }}{\mathrm{wrv}}_{i}\;A_{i}\overset{n}{\underset{i=1}{\sum }}{\mathrm{wcv}}_{i}\;A_{i}$ $=0{.73x}_{1}+0{.99x}_{2}+0{.95x}_{3}+1{.06x}_{4}+0{.77x}_{5}+0{.96x}_{6}+0{.83x}_{7}+0{.9x}_{8}+0{.36x}_{9}{+0x}_{10}$	WRV 10^4^ CNY wrv_i_ 10^4^ CNY/ha A_i_ ha

**Table 3:** Constraints and description of land use demand optimization model.

Constraint Condition	Formula
Total area constraint	$A=\overset{n}{\underset{i=1}{\sum }}A_{i}$ $=x_{1}{+x}_{2}{+x}_{3}{+x}_{4}{+x}_{5}{+x}_{6}{+x}_{7}{+x}_{8}{+x}_{9}{+x}_{10}=$ 1,876,897 ha
Forest cover constraint	1,876,897$\;\mathrm{ha}\;>\;x_{2}{+x}_{3}{+x}_{4}{+x}_{5}{+x}_{6}\;>\;$1,500,000 ha
Upper and lower boundaries of x_1_ to x_10_ in 2030	Lb = [128,000, 1,400,000, 20,000, 55,000, 0, 0, 0, 1000, 18,000, 23,000] ha Ub = [190,000, 1,500,000, 30,000, 65,000, 9000, 500, 68,000, 2,000, 19,000, 31,000] ha
Upper and lower boundaries of x_1_ to x_10_ in 2050	Lb = [128,000, 1,400,000, 26,000, 57,000, 0, 0, 0, 1000, 18,000, 23,000] ha Ub = [200,000, 1,876,897, 1,876,897, 1,876,897, 9000, 400, 70,000, 7000, 19,000, 60,000] ha

**Table 4:** Data sources and a detailed explanation of their usage.

Category	Data	Type	Source	Usage
Natural condition	DEM	Grid data	ASTER GDEM 30 m (http://www.gscloud.cn/sources/accessdata/310?pid=302, accessed on 5 June 2021)	Hydrological modeling/Driving factor of land use
	Soil	Grid data	HWSD v1.2 (http://www.fao.org/soils-portal/soil-survey/soil-maps-and-databases/harmonized-world-soil-database-v12/en/, accessed on 6 June 2021)	Hydrological modeling/Driving factor of land use
	Stream	Shapefile	Regional drainage map	Hydrological modeling/Driving factor of land use
	Weather data	ASCII text	http://data.cma.cn/, accessed on 1 June 2021	Hydrological modeling
	Precipitation	Grid data	WorldClim v2.0 (http://www.worldclim.org/, accessed on 1 June 2021)	Driving factor of land use
	Temperature	Grid data		Driving factor of land use
	Hydrologic data	ASCII text	Hydrological station	Validation of Hydrological Model
Social factor	Land use	Grid data	http://www.globallandcover.com/& http://maps.elie.ucl.ac.be/CCI/viewer/, accessed on 3 January 2021	Hydrological modeling/Multi-objective programming
	Government	Shapefile	https://www.tianditu.gov.cn/, accessed on 4 June 2021	Driving factor of land use
	GDP	Grid data	http://www.geodoi.ac.cn/WebCn/Default.aspx, accessed on 4 June 2021	Driving factor of land use
	Population	Grid data		Driving factor of land use
	Other data	ASCII text	Local government	Basic Parameters

**Table 5:** Water retention amount per unit area of different land use types.

Land Use Type	AGRL	FRSD	FRSE	FRSM	FRSO	SHRUB	GRAS	WETL	WATR	URBN
WL_i_ (10^4^ m^3^/ha)	0.244	0.332	0.316	0.355	0.258	0.321	0.275	0.300	−0.120	0

**Table 6:** Contribution grade standard of land use driving factors. X is the importance of the contribution of each driving factor, $\bar{X}$ is the mean of all data and σ is the standard deviation.

**Level**	**Low** **Contribution**	General Contribution	Medium Contribution	Great Contribution
Grading Standard	$0<x<\bar{X}-\sigma$	$\bar{X}-\sigma <x<\bar{X}$	$\bar{X}<x<\bar{X}+\sigma$	$\bar{X}+\sigma <x<1$
Grading Value	0–0.035	0.035–0.067	0.067–0.098	0.098–1
Amount of factors	19	67	46	18

**Table 7:** Land use demand forecast for 2030 and 2050. Land use code is the same as in Table 1.

Land Use Demand (ha)	2020	2030			2050		
		S1_ES	S2_ECS	S3_WCS	S1_ES	S2_ECS	S3_WCS
AGRL	190,014	191,000	182,035	180,439	200,000	140,007	158,637
FRSD	1,483,516	1,462,397	1,498,681	1,499,999	1,400,000	1,489,366	1,500,622
FRSE	26,472	30,000	23,710	26,799	26,000	44,750	68,734
FRSM	56,991	65,000	55,294	60,075	85,497	76,954	65,131
FRSO	8364	9000	3684	3208	9000	4098	771
SHRUB	347	500	274	169	400	253	274
GRASS	68,373	68,000	66,457	62,555	70,000	69,999	34,220
WETL	1054	2000	1217	1165	7000	4246	1698
WATR	18,751	19,000	18,257	18,670	19,000	18,001	18,010
URBN	23,758	31,000	27,288	23,818	60,000	29,223	28,800
EV (10^7^ CNY)	2908	3629	3241	2886	6541	3361	3293
ESV (10^7^ CNY)	6967	6950	6984	7006	6877	7094	7070
WRV (10^7^ CNY)	1773	1765	1773	1777	1735	1782	1782

## Data Availability

Not applicable.

## Appendix A

**Table A1:** The name and interpretation of the landscape pattern index used in the research.

Metrics	Explanation	Metrics	Explanation
PD	Patch Density	PLADJ	Proportion of Like Adjacencies Index
PAFRAC	Perimeter-Area Fractal Dimension	IJI	Interspersion and Juxtaposition Index
LSI	Landscape Shape Index	CONNECT	Landscape Connectivity Index
PLAND	Percentage of Landscape	COHESION	Patch Cohesion Index
COHESION	Patch Cohesion Index	DIVISION	Landscape Division Index
SHAPE	Mean Shape Index	SHDI	Shannon Diversity Index
LPI	Largest Patch Index	SIDI	Shannon’s Diversity Index
ED	Edge Density	SHEI	Shannon’s Evenness Index
CONTIG	Contiguity Index	AI	Aggregation Index
CONTAG	Contagion Index		

**Table A2:** Observed and simulated landscape pattern metrics of class-level.

Metric	PD			PAFRAC			LSI			PLAND			COHESION			AI		
	Ob10	Ob20	Sim20	Ob10	Ob20	Sim20	Ob10	Ob20	Sim20	Ob10	Ob20	Sim20	Ob10	Ob20	Sim20	Ob10	Ob20	Sim20
AGRL	0.15	0.17	0.32	1.35	1.31	1.34	96.4	101.1	128.7	11.4	10.1	10.2	99.4	99.3	99.3	93.8	93.1	91.2
FRSD	0.36	0.35	1.25	1.35	1.36	1.41	94.0	93.8	103.0	78.1	79.0	79.0	100.0	100.0	100.0	97.7	97.7	97.5
FRSE	0.01	0.01	0.07	1.30	1.25	1.14	14.7	14.8	17.4	1.4	1.4	1.4	99.6	99.6	99.5	97.4	97.4	97.0
FRSM	0.02	0.02	0.02	1.23	1.21	1.30	25.6	26.0	32.9	3.1	3.0	3.0	99.6	99.5	99.6	96.9	96.8	96.0
FRSO	0.10	0.10	0.11	1.29	1.27	1.32	57.3	53.6	60.6	0.3	0.4	0.4	88.8	91.6	91.6	78.3	82.7	80.4
SHRUB	0.01	0.01	0.01	1.28	1.28	1.30	15.3	14.7	16.6	0.0	0.0	0.0	84.6	86.4	85.2	75.4	77.5	74.4
GRASSS	4.07	3.97	4.18	1.43	1.45	1.45	285.9	286.5	294.5	3.9	3.6	3.6	95.7	95.8	95.8	68.3	67.2	66.3
WETL	0.00	0.00	0.00	1.21	1.30	1.23	3.9	8.2	7.0	0.0	0.1	0.0	96.5	98.3	96.2	94.7	93.3	92.8
WATR	0.12	0.12	0.14	1.54	1.54	1.54	59.0	59.8	60.5	1.0	1.0	1.0	99.4	99.4	99.4	87.2	87.1	86.9
URBN	0.03	0.04	0.08	1.22	1.30	1.24	27.6	44.8	41.9	0.8	1.3	1.3	97.1	97.6	97.0	93.3	91.5	92.0

**Table A3:** Observed and simulated landscape pattern metrics of landscape-level.

Metrics	Ob10	Ob20	Sim20	Metrics	Ob10	Ob20	Sim20
PD	4.86	4.79	6.18	CONNECT	0.04	0.04	0.05
SHAPE	1.24	1.26	1.22	COHESION	99.96	99.96	99.95
LPI	76.19	76.9	76.99	DIVISION	0.42	0.41	0.41
ED	27.44	27.66	30.6	SHDI	0.84	0.84	0.83
LSI	96.05	96.79	106.89	SIDI	0.37	0.36	0.36
CONTIG	0.23	0.22	0.18	SHEI	0.36	0.36	0.36
CONTAG	77.8	77.75	77.45	IJI	41.27	44.24	43.47
PLADJ	95.84	95.81	95.36	AI	95.88	95.85	95.4
